# Suppression of salivary melatonin secretion under 100-Hz flickering and non-flickering blue light

**DOI:** 10.1186/s40101-018-0183-9

**Published:** 2018-10-19

**Authors:** Tomoaki Kozaki, Yuki Hidaka, Jun-ya Takakura, Yosuke Kusano

**Affiliations:** 1 Department of Environmental Science, International College of Arts and Sciences, Fukuoka Women’s University, 1-1-1 Kasumigaoka, Higashi-ku, Fukuoka, 813-8529 Japan; 2 Faculty of Design, Kyushu University, Fukuoka, Japan; 3 Center for Social and Environmental Systems Research, National Institute for Environmental Studies, Tsukuba, Japan; 4 Department of Health and Nutrition Sciences, Nishikyushu University, Kanzaki, Japan

## Abstract

**Background:**

Bright light at night is known to suppress melatonin secretion. Novel photoreceptors named intrinsically photosensitive retinal ganglion cells (ipRGCs) are mainly responsible for projecting dark/bright information to the suprachiasmatic nucleus and thus regulating the circadian system. However, it has been shown that the amplitude of the electroretinogram of ipRGCs is considerably lower under flickering light at 100 Hz than at 1–5 Hz, suggesting that flickering light may also affect the circadian system. Therefore, in this study, we evaluated light-induced melatonin suppression under flickering and non-flickering light.

**Methods:**

Twelve male participants between the ages of 20 and 23 years (mean ± S.D. = 21.6 ± 1.5 years) were exposed to three light conditions (dim, 100-Hz flickering, and non-flickering blue light) from 1:00 A.M. to 2:30 A.M., and saliva samples were obtained just before 1:00 A.M. and at 1:15, 1:30, 2:00, and 2:30 A.M.

**Results:**

A repeated measures *t* test with Bonferroni correction showed that at 1:15 A.M., melatonin concentrations were significantly lower following exposure to non-flickering light compared with dim light, whereas there was no significant difference between the dim and 100-Hz flickering light conditions. By contrast, after 1:30 A.M., the mean melatonin concentrations were significantly lower under both 100-Hz flickering and non-flickering light than under dim light.

**Conclusion:**

Although melatonin suppression rate tended to be lower under 100-Hz flickering light than under non-flickering light at the initial 15 min of the light exposure, the present study suggests that 100-Hz flickering light may have the same impact on melatonin secretion as non-flickering light.

## Introduction

The development of artificial lighting systems has allowed us to have a bright environment at night, bringing comfort and safety. However, light has also been reported to have a range of physiological impacts on humans, such as changes in pupillary constriction [1–4], autonomic nervous function [5–7], brain activity [8], and cortisol secretion [9]. Furthermore, bright light at night is known to delay the circadian phase [10–12], resulting in disruption of the circadian rhythm [13], which, in turn, can lead to poor sleep and various kinds of health risks, such as cardiac disease and depression [14]. Bright light also suppresses the night-time secretion of the hormone melatonin [15–18], which is produced by the pineal gland and has several physiological activities, including anticancer activity [19–21]. As an example of the anticancer activity, Blask et al. [19] indicated that human breast cancer xenografts with melatonin-rich blood exhibited suppressed proliferative activity and linoleic acid uptake/metabolism, compared with melatonin-deficient blood. Consequently, the International Agency for Research on Cancer lists shiftwork that involves circadian disruption as a probable carcinogenic factor (Group 2A) and a prospective study has suggested that residential outdoor bright light at night may contribute to breast cancer risk [22]. Therefore, there is a real need to understand the physiological effects of light so that we can create healthy artificial lighting environments.

Novel retinal photoreceptors known as intrinsically photosensitive retinal ganglion cells (ipRGCs) or melanopsin-containing retinal ganglion cells (mRGCs) are mainly responsible for projecting dark/bright information to the suprachiasmatic nucleus and thus regulating the circadian system [23–26]. ipRGCs have several different properties from cones and rods, which are the traditional photoreceptors found in the eye. For example, the spectral peak sensitivity of ipRGCs is approximately 480 nm [23], resulting in an acute light-induced suppression of melatonin production in blue light [27–30]. Furthermore, the depolarizing voltage response of ipRGCs increases more slowly in response to a light pulse (i.e., has a long latency) and declines more slowly after the onset of the light pulse (i.e., exhibits sustained depolarization) compared with traditional photoreceptors [31]. In an examination of ipRGC electoretinograms (ERGs) under different light conditions, Takao et al. [32] found that ipRGCs that were exposed to flickering light at 100 Hz had steady-state ERGs, suggesting a high temporal frequency sensitivity, but lower-amplitude ERGs than those obtained under 1–5-Hz flickering light. Therefore, it appears that the flickering condition of light may also impact our circadian system.

In this study, we evaluated light-induced melatonin suppression under 100-Hz flickering and non-flickering blue light. We chose these conditions because ipRGCs are most sensitive to light at a wavelength of 480 nm and some artificial light sources such as fluorescent lamps flicker at approximately 100 Hz.

## Methods

### Subjects

Twelve Asian male subjects aged between 20 and 23 years (mean ± S.D. = 21.6 ± 1.5 years) participated in this study. Each subject gave informed consent to participate, had no history of psychiatric or sleep disorders, and was free from any medical conditions at the start of the experiment. The subjects were instructed to abstain from alcohol for 1 day and from caffeine and smoking for 3 h prior to the experiment. In addition, they were asked to keep a regular sleep-wake schedule (sleep onset between 1:00 and 2:00 A.M. and wake between 8:00 and 9:00 A.M.) for 6 days prior to the experiment. These sleep-wake schedules were checked by actigraphy devices (MotionWatch 8; CamNtech Ltd., Cambridge, UK) before each experiment.

### Experimental design

The experimental design followed the principles outlined in the Declaration of Helsinki and was approved by the ethical committee of the Faculty of Design, Kyushu University, Fukuoka, Japan. The experiments were carried out at the Research Center for Human Environmental Adaptation of Kyushu University between August and September 2016.

**Fig. 1:**
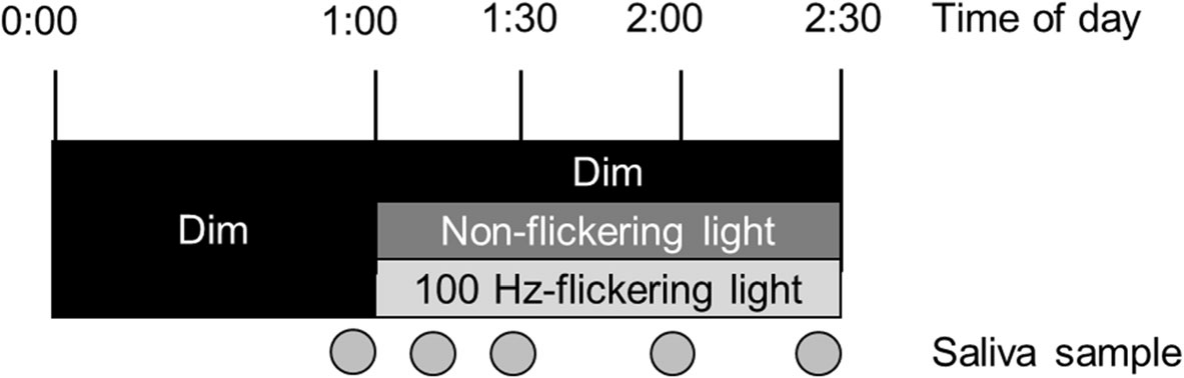
Experimental schedule

Subjects were exposed to three light conditions: dim (≤ 3 lx), 100-Hz flickering, and non-flickering blue light. Each light condition was provided in a random order at 6-day intervals. On the experimental day, each subject was admitted to the experimental chamber at 11:50 P.M. and a saliva sample was taken. The experiment started at 0:00 A.M. under the dim light condition. The experimental light condition was then provided for 1.5 h from 1:00 A.M. During the light exposure, the subjects remained awake and listened to music through headphones while sitting on a chair with their head resting on an ophthalmologic head holder to ensure delivery of the prescribed treatment. Saliva samples were taken just before 1:00 AM and then again at 1:15, 1:30, 2:00, and 2:30 A.M. The ambient temperature in the experimental chamber was maintained at 25 °C with a relative humidity of 50% (see Fig. 1 for an outline of the experimental schedule.)

### Light conditions

The 100-Hz flickering and non-flickering light conditions were delivered to each subject from a 0.5-m cubic light box. The two light conditions were obtained from arrays of blue light-emitting diodes (LEDs) on a board, which was placed with a white diffuser in front of the subject. The spectral emissions of the blue LEDs peaked at 465 nm, with a full width at half maximum of 24 nm. The 100-Hz flickering light was modulated by a pulse generator (PCI-6105; Interface Corp., Hiroshima, Japan) installed in a computer (AT-960, Epson Direct Corp., Matsumoto, Japan) with a purpose-built pulse width modulator circuit. The LED was on for 1 ms and off for 9 ms (10% duty ratio) in the flickering light condition. Before each experiment, the melanopic illuminance of the two light conditions at the subject’s cornea was adjusted to 38.0 μW/cm^2^ (irradiance was almost 52.0 μW/cm^2^) using a direct-current power source (KX-100H; Takasago Ltd., Kawasaki, Japan) and a spectroradiometer (LA-105; NK System, Osaka, Japan); Melanopic illuminance is a representation of irradiance by the activity of ipRGC [33]. The illuminance and photon density at the cornea were 30.2 lx and 14.09 log photons/s/cm^2^, respectively.

### Salivary melatonin sampling and analysis

Saliva samples were collected using non-cotton (polypropylene-polyethylene complex swab) collection devices (Sarstedt K.K., Numbrecht, Germany) to avoid any interference with the salivary melatonin assay results [34]. Saliva samples were centrifuged at 1500 *g* for 5 min and then frozen at − 30 °C until assay. Melatonin levels in the samples were analyzed using a commercially available radioimmunoassay (RIA) kit (Direct Saliva Melatonin RIA; Buhlmann Laboratories, Allschwil, Switzerland), which had a limit of detection of 0.9 pg/mL and a limit of quantification of 0.2 pg/mL. The mean intra- and inter-assay coefficients of variance were 7.9% and 9.8%, respectively.

### Data analysis

Given the substantial inter-individual variation in endogenous melatonin levels [35, 36], melatonin suppression rates were calculated using the following formula:

Melatonin suppression = (concentration at each time point after light exposure − concentration before light exposure)/concentration before light exposure.

Differences in the mean melatonin concentration and melatonin suppression rate over time and between light conditions were analyzed using a two-way within-subject repeated measures analysis of variance with the Huynh-Feldt *ε* correction, to evaluate *F* ratios for repeated measures involving more than one degree of freedom. Post hoc analyses were then conducted using repeated measures *t* tests with the Bonferroni correction for the mean melatonin concentration and repeated measures *t* tests for the melatonin suppression rate. All statistical analyses were performed in SPSS (version 22.0; SPSS, Chicago, US) with a significance level of *p* < 0.05.

## Results

Data from one subject were excluded from the analysis because his melatonin levels were lower than the limit of detection of the RIA kit (0.9 pg/ml). Therefore, data from 11 subjects are reported below.

**Fig. 2:**
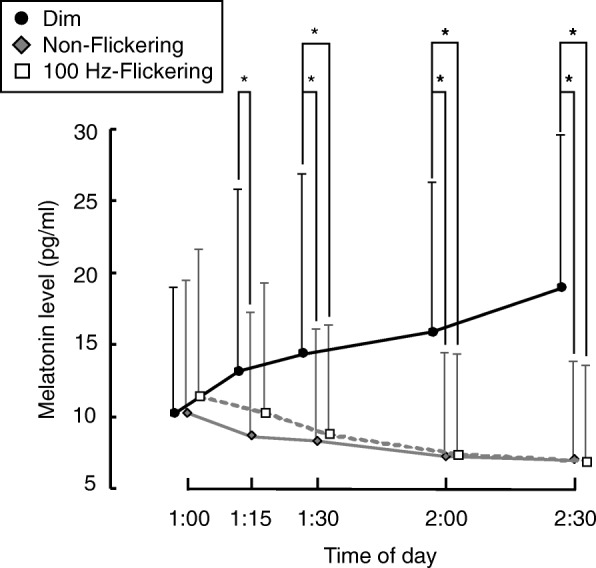
Temporal changes in the mean melatonin concentrations under dim (dark circle), non-flickering (gray diamond), and 100-Hz flickering blue light (white square). Data are shown as mean + S.D. A number of the subject was eleven. Single asterisk (*) means statistical significance (*p* < 0.05) between light conditions by a repeated measures *t* test with the Bonferroni correction

Temporal changes in the mean melatonin concentrations under the three light conditions are shown in Fig. 2. The light condition had a significant effect on melatonin concentration (*F*_2.20_ = 21.00, *p* < 0.01, *ε* = 0.66), and there was a significant interaction between the time interval and light condition (*F*_8.80_ = 11.75, *p* < 0.01, *ε* = 0.30). Post hoc comparisons showed that the melatonin concentration was significantly lower under non-flickering light than under dim light at 1:15 A.M., whereas there was no significant difference between the concentration under dim light and 100-Hz flickering light. However, after 1:30 A.M., the mean melatonin concentrations were significantly lower under both 100-Hz flickering and non-flickering light than under dim light (Fig. 2).

**Fig. 3:**
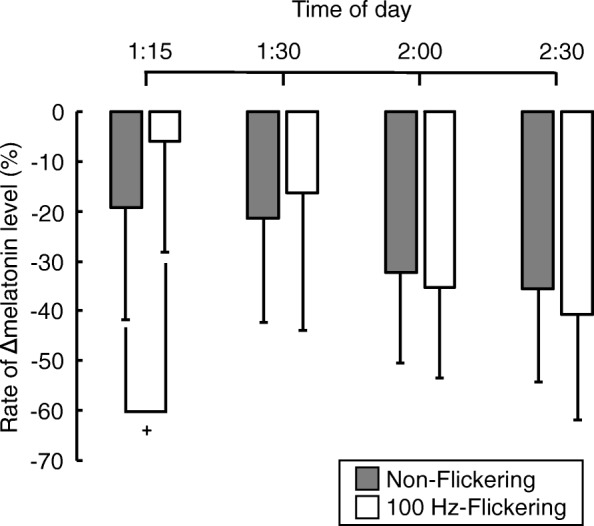
Temporal changes in the rate of melatonin suppression under non-flickering (gray bar) and 100-Hz flickering blue light (white bar). Data are shown as mean + S.D. A number of the subject was eleven. Single plus (+) means statistical tendency (*p* < 0.1) between non-flickering and 100 Hz-flickering light condition by a repeated measures *t* test

Temporal changes in the melatonin suppression rate under the three light conditions are shown in Fig. 3. Time interval had a significant effect on the melatonin suppression rate (*F*_3.30_ = 11.88, *p* < 0.01, *ε* = 0.50), and there was a significant interaction between the time interval and light condition (*F*_3.30_ = 4.01, *p* < 0.05, *ε* = 1.00). The melatonin suppression rate tended to be lower under 100-Hz flickering light than under non-flickering light at 1:15 A.M. However, after 1:30 AM, there was no significant difference in the melatonin suppression rate between the 100-Hz flickering and non-flickering light conditions (Fig. 3).

## Discussion

Melatonin suppression rate under 100-Hz flickering light showed a tendency to be lower than under non-flickering light 15 min after the start of the experiment, despite having no significant difference on mean melatonin concentration between the light conditions. This finding suggests a capability that the high-frequency flickering and non-flickering light may have a different impact on melatonin secretion. From a physiological perspective, this may be explained by the temporal properties of ipRGCs. Takao et al. [32] obtained larger-amplitude ERGs from mouse retinae under 1–5-Hz flickering light than under 100-Hz flickering light. Therefore, it is possible that the amplitude of ERGs under non-flickering light may also be larger than under 100-Hz flickering light. Another explanation may be the rapidity of adaptation of ipRGCs to the light conditions. It has previously been reported that the ERGs of ipRGCs are significantly larger in dark-adapted retinae than in light-adapted retinae [32]. In the present study, the subjects spent 1 h in dim light conditions (≤ 3 lx) before the light exposure, allowing their retinae to adapt to this. Therefore, although we have no conclusive evidence, there may be a difference in the rapidity of adaptation of ipRGCs to 100-Hz flickering and non-flickering light.

These findings indicated a small difference in mean melatonin concentration between 100-Hz flickering and non-flickering light at 1:15 A.M., which may have health benefits given the anticancer activity of melatonin. However, the efficacy of this treatment may be limited, as we only observed a difference in melatonin suppression between 100-Hz flickering and non-flickering light at 15 min after the light exposure, indicating that the ipRGCs may respond to 100-Hz flickering light in the same manner as to non-flickering light but after a delay of several minutes. In addition, 100-Hz flickering light may not prevent a shift in the circadian phase, as it has previously been found that a sequence of 2-ms flashes of bright light occurring once per minute for 60 min delays the melatonin onset time, despite having no effect on melatonin secretion [37], and that 2-ms light flashes change the circadian phase even while the subjects are asleep [38]. Therefore, it appears that circadian phase shifting is more sensitive to flickering light than melatonin suppression.

Although this study suggests that high-frequency flickering light may have less impact on melatonin secretion than non-flickering light, the difference was small and limited. However, the effect of 100-Hz flickering light on melatonin secretion may be related to the light intensity. It has been shown that ipRGCs show continued discharge after the light offset following exposure to bright light, whereas no such effect occurs at lower light levels [31], and the amplitude of the ERG response of ipRGCs between dark-adapted and light-adapted retinae differs more greatly at higher light levels than at lower light levels [32]. Therefore, further research is needed to determine how best to create a healthy artificial lighting environment.

## Conclusions

This study evaluated light-induced melatonin suppression under 100-Hz flickering and non-flickering blue light. The melatonin concentration was significantly lower under non-flickering light than under dim light 15 min after the light exposure, whereas there was no significant difference between the concentration under dim light and 100-Hz flickering light. These findings suggest that 100-Hz flickering light may have the same impact on melatonin secretion as non-flickering light.

## Abbreviations

ERGs: Electoretinograms
ipRGCs: Intrinsically photosensitive retinal ganglion cells
LEDs: Light-emitting diodes
mRGCs: Melanopsin-containing retinal ganglion cells
RIA: Radioimmunoassay

## References

[1] Lee S, Ishibashi S, Shimomura Y, Katsuura T (2016). Effect of simultaneous exposure to extremely short pulses of blue and green light on human pupillary constriction. J Physiol Anthropol.

[2] Lee S, Muto N, Shimomura Y, Katsuura T (2017). Human pupillary light reflex during successive irradiation with 1-ms blue- and green-pulsed light. J Physiol Anthropol.

[3] Lee S, Uchiyama Y, Shimomura Y, Katsuura T (2017). Subadditive responses to extremely short blue and green pulsed light on visual evoked potentials, pupillary constriction and electroretinograms. J Physiol Anthropol.

[4] Daneault V, Dumont M, Masse E, Vandewalle G, Carrier J (2016). Light-sensitive brain pathways and aging. J Physiol Anthropol.

[5] Yuda E, Ogasawara H, Yoshida Y, Hayano J (2016). Suppression of vagal cardiac modulation by blue light in healthy subjects. J Physiol Anthropol.

[6] Yuda E, Ogasawara H, Yoshida Y, Hayano J (2017). Exposure to blue light during lunch break: effects on autonomic arousal and behavioral alertness. J Physiol Anthropol.

[7] Yuda E, Ogasawara H, Yoshida Y, Hayano J (2017). Enhancement of autonomic and psychomotor arousal by exposures to blue wavelength light: importance of both absolute and relative contents of melanopic component. J Physiol Anthropol.

[8] Dai Q, Uchiyama Y, Lee S, Shimomura Y, Katsuura T (2017). Effect of quantity and intensity of pulsed light on human non-visual physiological responses. J Physiol Anthropol.

[9] Adamsson M, Laike T, Morita T (2016). Annual variation in daily light exposure and circadian change of melatonin and cortisol concentrations at a northern latitude with large seasonal differences in photoperiod length. J Physiol Anthropol.

[10] Czeisler CA, Allan JS, Strogatz SH, Ronda JM, Sanchez R, Rios CD, Freitag WO, Richardson GS, Kronauer RE (1986). Bright light resets the human circadian pacemaker independent of the timing of the sleep-wake cycle. Science.

[11] Lockley SW, Brainard GC, Czeisler CA (2003). High sensitivity of the human circadian melatonin rhythm to resetting by short wavelength light. J Clin Endocrinol Metab.

[12] Smith MR, Eastman CI (2009). Phase delaying the human circadian clock with blue-enriched polychromatic light. Chronobiol Int.

[13] Figueiro MG (2017). Disruption of circadian rhythms by light during day and night. Curr Sleep Med Rep.

[14] Lunn RM, Blask DE, Coogan AN, Figueiro MG, Gorman MR, Hall JE, Hansen J, Nelson RJ, Panda S, Smolensky MH (2017). Health consequences of electric lighting practices in the modern world: a report on the National Toxicology Program’s workshop on shift work at night, artificial light at night, and circadian disruption. Sci Total Environ.

[15] Lewy AJ, Wehr TA, Goodwin FK, Newsome DA, Markey SP (1980). Light suppresses melatonin secretion in humans. Science.

[16] Brainard GC, Lewy AJ, Menaker M, Fredrickson RH, Miller LS, Weleber RG, Cassone V, Hudson D (1988). Dose-response relationship between light irradiance and the suppression of plasma melatonin in human volunteers. Brain Res.

[17] McIntyre IM, Norman TR, Burrows GD, Armstrong SM (1989). Human melatonin suppression by light is intensity dependent. J Pineal Res.

[18] Aoki H, Yamada N, Ozeki Y, Yamane H, Kato N (1998). Minimum light intensity required to suppress nocturnal melatonin concentration in human saliva. Neurosci Lett.

[19] Blask DE, Brainard GC, Dauchy RT, Hanifin JP, Davidson LK, Krause JA, Sauer LA, Rivera-Bermudez MA, Dubocovich ML, Jasser SA (2005). Melatonin-depleted blood from premenopausal women exposed to light at night stimulates growth of human breast cancer xenografts in nude rats. Cancer Res.

[20] Stevens RG, Blask DE, Brainard GC, Hansen J, Lockley SW, Provencio I, Rea MS, Reinlib L (2007). Meeting report: the role of environmental lighting and circadian disruption in cancer and other diseases. Environ Health Perspect.

[21] Stevens RG, Brainard GC, Blask DE, Lockley SW, Motta ME (2014). Breast cancer and circadian disruption from electric lighting in the modern world. CA Cancer J Clin.

[22] James P, Bertrand KA, Hart JE, Schernhammer ES, Tamimi RM, Laden F (2017). Outdoor light at night and breast cancer incidence in the Nurses’ Health Study II. Environ Health Perspect.

[23] Berson DM, Dunn FA, Takao M (2002). Phototransduction by retinal ganglion cells that set the circadian clock. Science.

[24] Hattar S, Liao HW, Takao M, Berson DM, Yau KW (2002). Melanopsin-containing retinal ganglion cells: architecture, projections, and intrinsic photosensitivity. Science.

[25] Hattar S, Lucas RJ, Mrosovsky N, Thompson S, Douglas RH, Hankins MW, Lem J, Biel M, Hofmann F, Foster RG (2003). Melanopsin and rod-cone photoreceptive systems account for all major accessory visual functions in mice. Nature.

[26] Lucas RJ, Hattar S, Takao M, Berson DM, Foster RG, Yau KW (2003). Diminished pupillary light reflex at high irradiances in melanopsin-knockout mice. Science.

[27] Brainard GC, Hanifin JP, Greeson JM, Byrne B, Glickman G, Gerner E, Rollag MD (2001). Action spectrum for melatonin regulation in humans: evidence for a novel circadian photoreceptor. J Neurosci.

[28] Thapan K, Arendt J, Skene DJ (2001). An action spectrum for melatonin suppression: evidence for a novel non-rod, non-cone photoreceptor system in humans. J Physiol.

[29] Kozaki T, Koga S, Toda N, Noguchi H, Yasukouchi A (2008). Effects of short wavelength control in polychromatic light sources on nocturnal melatonin secretion. Neurosci Lett.

[30] Brainard GC, Hanifin JP, Warfield B, Stone MK, James ME, Ayers M, Kubey A, Byrne B, Rollag M (2015). Short-wavelength enrichment of polychromatic light enhances human melatonin suppression potency. J Pineal Res.

[31] Dacey DM, Liao HW, Peterson BB, Robinson FR, Smith VC, Pokorny J, Yau KW, Gamlin PD (2005). Melanopsin-expressing ganglion cells in primate retina signal colour and irradiance and project to the LGN. Nature.

[32] Takao M, Fukuda Y, Morita T (2017). A novel intrinsic electroretinogram response in isolated mouse retina. Neuroscience.

[33] Lucas RJ, Peirson SN, Berson DM, Brown TM, Cooper HM, Czeisler CA, Figueiro MG, Gamlin PD, Lockley SW, O'Hagan JB (2014). Measuring and using light in the melanopsin age. Trends Neurosci.

[34] Kozaki T, Hidaka Y (2018). Non-cotton swab sample collection may not affect salivary melatonin assay results. J Physiol Anthropol.

[35] Arendt J (1998). Melatonin and the pineal gland: influence on mammalian seasonal and circadian physiology. Rev Reprod.

[36] Burgess HJ, Fogg LF (2008). Individual differences in the amount and timing of salivary melatonin secretion. PLoS One.

[37] Zeitzer JM, Ruby NF, Fisicaro RA, Heller HC (2011). Response of the human circadian system to millisecond flashes of light. PLoS One.

[38] Zeitzer JM, Fisicaro RA, Ruby NF, Heller HC (2014). Millisecond flashes of light phase delay the human circadian clock during sleep. J Biol Rhythm.

